# Prevalence of polypharmacy and associated adverse health outcomes in adult patients with chronic kidney disease: protocol for a systematic review and meta-analysis

**DOI:** 10.1186/s13643-021-01752-z

**Published:** 2021-07-04

**Authors:** Ikechi G. Okpechi, Mohammed M. Tinwala, Shezel Muneer, Deenaz Zaidi, Feng Ye, Laura N. Hamonic, Maryam Khan, Naima Sultana, Scott Brimble, Allan Grill, Scott Klarenbach, Cliff Lindeman, Amber Molnar, Dorothea Nitsch, Paul Ronksley, Soroush Shojai, Boglarka Soos, Navdeep Tangri, Stephanie Thompson, Delphine Tuot, Neil Drummond, Dee Mangin, Aminu K. Bello

**Affiliations:** 1 Department of Medicine, University of Alberta, Edmonton, AB Canada; 2 Division of Nephrology, University of Cape Town, Cape Town, South Africa; 3 John W. Scott Health Sciences Library, University of Alberta, Edmonton, Alberta Canada; 4 Faculty of Science, University of Alberta, Edmonton, Alberta Canada; 5 Division of Nephrology, Department of Medicine, McMaster University, Hamilton, ON Canada; 6 Department of Family and Community Medicine, University of Toronto, Toronto, ON Canada; 7 Department of Family Medicine, University of Alberta, Edmonton, AB Canada; 8 Division of Nephrology, Department of Medicine, McMaster University, Hamilton, Ontario Canada; 9 Department of Health Research Methods, Evidence, and Impact, McMaster University, Hamilton, Ontario Canada; 10 Department of Non-Communicable Disease Epidemiology, London School of Hygiene and Tropical Medicine, London, UK; 11 Department of Community Health Sciences, University of Calgary, Calgary, AB Canada; 12 Department of Family Medicine, University of Calgary, Calgary, AB Canada; 13 Department of Medicine, Max Rady College of Medicine, Winnipeg, MB Canada; 14 Division of Nephrology, University of California, San Francisco, CA USA; 15 Kidney Health Research Institute, University of California, San Francisco, CA USA; 16 Department of Family Medicine, McMaster University, Hamilton, ON Canada; 17 Division of Nephrology and Immunology, Faculty of Medicine and Dentistry, University of Alberta, Edmonton, Alberta Canada

**Keywords:** CKD, Polypharmacy, Elderly, Multimorbidity, Adverse effects, Prescriptions

## Abstract

**Background:**

Polypharmacy, often defined as the concomitant use of ≥ 5 medications, has been identified as a significant global public health threat. Aging and multimorbidity are key drivers of polypharmacy and have been linked to a broad range of adverse health outcomes and mortality. Patients with chronic kidney disease (CKD) are particularly at high risk of polypharmacy and use of potentially inappropriate medications given the numerous risk factors and complications associated with CKD. The aim of this systematic review will be to assess the prevalence of polypharmacy among adult patients with CKD, and the potential association between polypharmacy and adverse health outcomes within this population.

**Methods/design:**

We will search empirical databases such as MEDLINE, Embase, Cochrane Library, CINAHL, Web of Science, and PsycINFO and grey literature from inception onwards (with no language restrictions) for observational studies (e.g., cross-sectional or cohort studies) reporting the prevalence of polypharmacy in adult patients with CKD (all stages including dialysis). Two reviewers will independently screen all citations, full-text articles, and extract data. Potential conflicts will be resolved through discussion. The study methodological quality will be appraised using an appropriate tool. The primary outcome will be the prevalence of polypharmacy. Secondary outcomes will include any adverse health outcomes (e.g., worsening kidney function) in association with polypharmacy. If appropriate, we will conduct random effects meta-analysis of observational data to summarize the pooled prevalence of polypharmacy and the associations between polypharmacy and adverse outcomes. Statistical heterogeneity will be estimated using Cochran’s Q and I^2^ index. Additional analyses will be conducted to explore the potential sources of heterogeneity (e.g., sex, kidney replacement therapy, multimorbidity).

**Discussion:**

Given that polypharmacy is a major and a growing public health issue, our findings will highlight the prevalence of polypharmacy, hazards associated with it, and medication thresholds associated with adverse outcomes in patients with CKD. Our study will also draw attention to the prognostic importance of improving medication practices as a key priority area to help minimize the use of inappropriate medications in patients with CKD.

**Systematic review registration:**

PROSPERO registration number: [CRD42020206514].

**Supplementary Information:**

The online version contains supplementary material available at 10.1186/s13643-021-01752-z.

## Background

Polypharmacy, the concurrent use of multiple medications by an individual, is a significant and growing public health threat worldwide [1–3]. In 2017, the World Health Organization (WHO) highlighted polypharmacy as a key action to address unsafe medication practices and medication errors which are often associated with significant patient injury and preventable harm [1]. Globally, the prevalence of polypharmacy continues to increase, with estimates of 10–20% in the general population and 40–60% in the elderly [4–6]. There is no standard definition of polypharmacy; various definitions and related terms have been used (reviewed in detail by Masnoon et al.) [7]. Variability in the numerical threshold used in characterizing polypharmacy, as well as the reported inconsistencies regarding duration of therapy, healthcare setting, and inclusion of over-the-counter (OTC), and traditional and complementary medicines often creates challenges when defining polypharmacy [7]. Most studies, however, define polypharmacy as the concomitant use of ≥ 5 medications which is supported by evidence on medication-related adverse effects associated with polypharmacy [8].

Aging and multimorbidity are the key driving factors of polypharmacy [2, 3]. It is estimated that about half of individuals over the age of 65 years have at least three coexisting chronic conditions, and about 20% have five or more [ 9]. Given a rising burden of multimorbidity among the elderly, there is a corresponding increase in the amount of medication use in this group, thus increasing the prevalence of polypharmacy [2, 3]. In the USA, more than 4 in 10 older adults take 5 or more prescription medications daily—a marked rise of 300% from 1994 to 2014 [10]. This proportion increases to 7 in 10 older adults if OTC medications and supplements are counted [11] and nearly 20% of older adults take ≥ 10 drugs [10]. Similarly, the Canadian Institute for Health Information (CIHI) have reported that almost two-thirds of individuals aged 65 years and over used ≥ 5 drug classes, while 27% ≥ 10 and 8.6% used ≥ 15 [12]. Table 1 summarizes other driving forces of polypharmacy.

**Table 1:** Drivers of Polypharmacy and Various Associated Negative Consequences

Drivers of Polypharmacy	Negative Consequences Associated with Polypharmacy [13–16]
i. Indiscriminate use of clinical practice guidelines (CPGs) designed for the management of single diseases in elderly, multimorbid patients [17, 18].	i. Adverse drug events (ADEs) ii. Adverse drug reactions (ADRs) iii. Drug-drug interactions iv. Drug-disease interactions v. Medications non-adherence vi. Medication errors vii. Use of potentially inappropriate medications (PIMs) viii. Renal failure ix. Urinary incontinence x. Falls and fractures xi. Functional decline, disability and frailty xii. Cognitive impairment and delirium xiii. Malnutrition xiv. Decreased quality of life xv. Nursing home/long-term care placement xvi. Hospitalizations xvii. Mortality
ii. Protocol-driven medicine that recommends prescribing medications as the first line of treatment and “stepping up” drug regimens with higher doses and/or additional drugs if targets are not reached [17, 19, 20].	
iii. Performance standards and incentives that coerce clinicians to follow guidelines focused on starting medications [18, 19].	
iv. Research gaps that leaves many aspects of polypharmacy poorly understood [18, 19].	
v. Inadequate clinician training on the management of polypharmacy (including monitoring, detecting, preventing, and evaluating adverse outcomes associated with polypharmacy) [18, 19].	
vi. Fragmented healthcare systems that results in uncoordinated treatment by multiple prescribers [9, 19, 21].	
vii. Growth of the pharmaceutical industry leading to an ever-increasing availability of medications for a growing number of medical conditions [19].	
viii. Influence of the pharmaceutical industry on clinicians [19] and funding of clinical drug trials [22] and professional societies that publish CPGs [23].	

Polypharmacy is associated with a broad range of adverse health outcomes, resulting in significant costs to both the patient and the healthcare system [13–15], with the risk and severity of harm increasing with an increasing number of medications [16]. Polypharmacy has also been strongly associated with adverse drug events (ADEs), adverse drug reactions (ADRs), drug-drug interactions, and drug-disease interactions [13–15]. In the USA, ADRs are estimated to claim between 100,000 and 218,000 lives annually and cost the healthcare system up to $137–177 billion [24]. The true incidence of ADRs are estimated to be much higher since underreporting is shown to be as high as 94% [25]. Studies have also shown that the risk of ADEs increases by up to 7–10% for each additional medication taken, and that outpatients using 5 or more medications have an 88% increased chance of experiencing an ADE compared to those taking less medications [26].

Polypharmacy has also been directly associated with mortality. In a systematic review that investigated the association between polypharmacy and mortality, a significant association between polypharmacy and death was observed when polypharmacy was defined as a discrete variable (pooled-adjusted odds ratio [aOR] 1.08 [95% CI 1.04–1.12]) [27]. When polypharmacy was defined categorically, a dose-response relationship was also observed across increasing thresholds of medications use, i.e., 1–4 medications [aOR 1.24]; 5 medications [aOR 1.31] and 6–9 medications [aOR 1.59]. Excessive polypharmacy (i.e., use of ≥ 10 medications) was also significantly associated with death [aOR 1.96] [27]. Other negative consequences associated with polypharmacy are also summarized in Table 1 [13–23].

An increasingly aging and multimorbid population has contributed to the rising prevalence of chronic kidney disease (CKD) [28, 29]. Individuals with CKD are highly vulnerable to polypharmacy given that CKD risk factors (e.g., diabetes mellitus and hypertension) and other cardiovascular diseases and risk factors are more prevalent in this population [30, 31]. Furthermore, patients with CKD require additional medications to limit the progression of CKD and manage disease-related complications (e.g., anemia, metabolic disorders, hyperlipidemia, mineral, and bone disorders) as kidney function deteriorates [32]. In CKD, all mechanisms of kidney excretion are impaired, including glomerular filtration, tubular secretion, and reabsorption. Since most drugs are largely eliminated through the kidneys, reduced kidney function causes a wide-ranging changes to the pharmacokinetic (drug absorption, distribution, metabolism, and excretion) and pharmacodynamic (drug-receptor interactions) properties of drugs thereby increasing the likelihood of potentially life-threatening toxicities, drug-drug interactions, and ADRs [33, 34]. The substantial burden of medications in individuals with CKD is well-documented, with studies showing that between 70.4–81% of patients with CKD take ≥ 5 medications [35, 36]. In a German study of 5217 patients with CKD, the prevalence of polypharmacy at baseline and follow-up was almost 80%, the median number of different medications per day was 8 (range 0–27), and factors associated with polypharmacy were increasing CKD stage, age, body mass index (BMI), diabetes mellitus, cardiovascular disease, and a history of smoking [35]. Another study found that 35.5% of older individuals with CKD took ≥ 10 medications, vitamins, and supplements [36].

Polypharmacy is also the main reason for potentially inappropriate medications (PIMs) use, with the number of PIMs increasing with the number of prescribed medications [37]. In individuals with CKD, numerous studies have revealed that PIM use as well as renally inappropriate medications use (RIMs) is common [36–39] and often overlooked [38, 39]. The prevalence of PIMs is estimated to be as much as 62–67% of patients in the inpatient and ambulatory care setting [40]. The recently published CKD-REIN study revealed that a large proportion of medications prescribed to patients with CKD were contraindicated, suggesting a lack of regular and/or thorough assessment of patients’ medication lists as kidney function declined [41].

The number of older persons is expected to have doubled by 2050 (reaching nearly 2.1 billion) [42], and this is likely to further increase the burden of multimorbidity and prevalence of individuals with CKD on polypharmacy [28]. Given the relationship between multimorbidity and CKD, and that such patients are at high risk of polypharmacy, PIMs, and adverse health effects associated with polypharmacy, this study will provide adequate information characterizing the burden of these conditions among patients with CKD. This will likely improve caution when prescribing medications for patients with CKD and reduce the frequency of adverse health effects related to polypharmacy in this population.

The aim of this systematic review will be to assess the prevalence of polypharmacy among adult patients with CKD, and the potential association between polypharmacy and adverse health outcomes within this population.

## Methods/design

The present protocol has been registered within the PROSPERO database (registration number CRD42020206514) and is being reported in accordance with the guidance provided in the Preferred Reporting Items for Systematic Reviews and Meta-Analyses Protocols (PRISMA-P) statement [43] (see checklist in Additional file 1). The planned review will be reported according to the Preferred Reporting Items for Reporting Systematic Reviews and Meta-Analyses (PRISMA) 2020 Statement [44], and the Meta-analysis Of Observational Studies in Epidemiology (MOOSE) reporting guideline [45].

### Information sources and search strategies

The following databases will be searched from inception onwards with no language restriction: MEDLINE, Embase, Cochrane Library, CINAHL, Web of Science, and PsycINFO. The search strategy will be developed in consultation with a research librarian, the study investigators, and with guidance from the Cochrane handbook. We will also use a combination of controlled vocabulary search terms (i.e., Medical Subject Headings), and we will adapt the MEDLINE search strategy for the other databases. The draft search strategy for MEDLINE is provided in Additional file 2.

Reference lists of all relevant and selected publications will be searched manually to identify further studies. Conference abstracts and grey literature studies will also be considered from recommended resources in consultation with our medical librarian. As such, we will search ProQuest Dissertations & Theses Global, and Conference Proceedings Citation Index (Clarivate Analytics).

### Criteria for considering studies for the review

#### Types of studies

Eligible studies will be observational studies (cohort, cross-sectional, or health surveys) reporting prevalence data of polypharmacy. Cross-sectional studies will be the most appropriate study design to determine the prevalence of polypharmacy. Cross-sectional health surveys are typically used to estimate the point prevalence of common conditions of long duration.

#### Types of participants

We will include studies with participants over 18 years of age, regardless of sex and ethnicity, across the spectrum of CKD (including those receiving kidney replacement therapy), and studies performed in in-patient and outpatient settings. We will exclude studies where the prevalence of polypharmacy cannot be computed as well as review articles, case reports, case studies, images, and other studies from which relevant data cannot be obtained even after attempting to contact the authors.

#### Exposure of interest

We will include studies that explored polypharmacy in patients with CKD and reported the prevalence of polypharmacy in their study setting with or without reporting the duration of therapy and/or healthcare setting (e.g., ambulatory care, hospitalization). We will define polypharmacy as the use of ≥ 5 medications (i.e., prescriptions, not pills, or dispensations) and excessive polypharmacy (hyperpolypharmacy) as use of ≥ 10 medications.

#### Types of outcome

The primary outcome will be the prevalence of polypharmacy (as described above). Secondary outcomes will include any adverse health outcomes in association with polypharmacy. Specifically, we will include studies that reported an association between polypharmacy and the following adverse health outcomes: kidney-related outcomes (such as episodes of acute kidney injury, worsening of estimated glomerular filtration rate [eGFR], initiation of dialysis, episodes of hyperkalemia), cardiovascular-related outcomes (such as fatal and non-fatal cardiovascular events), all-cause mortality, hospitalizations, drug-related outcomes (ADRs, ADEs, etc.), health-related quality of life (HRQoL), and other cognitive and physical decline outcomes.

### Data collection and analysis

#### Study selection

A flow diagram showing details of studies included and excluded at each stage of the study selection process will be provided in the final review. We will adopt a 2-stage collaborative review process for screening and inclusion of studies. First, 2 investigators (MT and SM) will independently review the titles and abstracts of retrieved studies using the established study inclusion and exclusion criteria. Subsequently, full texts of the selected studies will be obtained and will be independently assessed by these reviewers for eligibility. If necessary, any discrepancies will be resolved by a third reviewer (IGO). For any excluded study, one of the predefined exclusion criteria will be recorded.

#### Data extraction and management

Two investigators will independently extract data from selected studies. The extracted data will be entered into a standardized form and will be reviewed for accuracy and completeness. Data elements from the selected studies will include first author, country, year of publication, study design, sample size, age, sex, CKD stages, and/or kidney function (i.e., serum creatinine and/or estimated glomerular filtration rate), number of comorbidities, duration of therapy, healthcare setting, mean/median number of medications, the article’s definition of polypharmacy, prevalence of polypharmacy, and adverse health outcomes reported. Reported adverse health outcomes will be grouped into ADRs, ADEs, or others and recorded as reported from the studies. Authors of the studies will be contacted for missing or additional data. If possible, we will calculate missing data using available information (e.g., imputation). All missing data will be reported in the data extraction form and risk of bias table.

#### Assessment of risk of bias

Methodological quality of the studies will be evaluated using the checklist developed by Hoy et al. [46] to assess the risk of bias from studies. This quality assessment tool incorporates assessments of risk of bias across core domains including sampling, the sampling technique and size, outcome measurement, response rate, and statistical reporting. The overall risk of bias for each study will be displayed in a risk of bias summary table.

#### Data synthesis

Study characteristics will be summarized in Tables and described in texts in the study manuscript.. The decision to perform a meta-analysis on the primary outcome (prevalence of polypharmacy) will depend on the assessment of statistical heterogeneity. If heterogeneity between studies is high (*I*^2^ > 50% and deemed to represent considerable heterogeneity), then data will be reported descriptively and we will provide a narrative synthesis of included studies using the Synthesis Without Meta-analysis (SWiM) reporting guideline as a framework [47]. If heterogeneity is acceptable, we will pool the study-specific estimates using a random-effects meta-analysis model (DerSimonian and Laird) to obtain an overall summary estimate of the prevalence of polypharmacy and rates of the secondary outcomes [48] [49].Heterogeneity among studies will be evaluated in relation to participant characteristics (e.g., comorbidities), number of medications, and types of adverse health outcomes (kidney-related/others). We will evaluate heterogeneity across studies by applying the χ2 test (p < 0.1 will indicate heterogeneity) and quantified using the I^2^ statistic (values of < 25%, 25–75%, and > 75% representing low, medium, and high heterogeneity, respectively) [50]. We will assess the presence of publication bias using funnel plots and Egger’s test [51].

### Additional analyses

Subgroup analysis will be performed to investigate variations in prevalence data and potential sources of heterogeneity [49]. The following subgroups will be considered for further analyses: sex (males versus females), age groups: elderly and very elderly, study setting (in-patient versus outpatient settings), quality of study (low versus moderate versus high), patients on kidney replacement therapy (KRT) versus not receiving KRT, and multimorbidity (≤ 3 versus > 3). Significant results will be defined as p < 0.05.

#### Confidence in cumulative evidence

The quality of evidence for the primary outcome, i.e., prevalence of polypharmacy, will be assessed as “very low” to “high” in accordance to the Grading of Recommendations Assessment, Development and Evaluation (GRADE) Workgroup [52]. We will identify limitations of included studies and suggest improvement where possible.

#### Patient and public involvement

Given that this study is a systematic review, there will be no direct involvement of patients and public.

#### Ethics and dissemination

Ethical approval will not be required for this study as data used will be extracted from published studies. Results of this review will be published using traditional approaches including open-access peer-reviewed publications(s), presentations at relevant scientific conferences, reports, and lay summaries.

## Discussion

To the best of our knowledge, this review is the first to comprehensively assess the prevalence of polypharmacy in patients with CKD as well as reporting the incidence of adverse health outcomes associated with polypharmacy in such patients. As polypharmacy is a major and growing public health issue, our findings will highlight the hazards associated with polypharmacy as well as medication thresholds associated with adverse outcomes. Our study will also draw attention to the prognostic importance of improving medication practices as a key priority area to help minimize the use of inappropriate medications in patients with CKD. A potential limitation of this study could be non-uniform reporting of adverse outcomes and their associations with polypharmacy. This could make it difficult to identify adverse outcomes associated with use of medications in the CKD population. Another limitation could be that identified studies are mostly of low quality which could impact on the final reporting of our outcomes. Despite these, we expect that the results of this study will inform medication education, physician practice guidelines, and various quality improvement initiatives to address polypharmacy. Any amendments we make to this protocol when conducting the review will be outlined in PROSPERO and reported in the final manuscript.

## Supplementary Information

**Additional file 1:.** PRISMA-P checklist.

**Additional file 2:.** Ovid MEDLINE search terms and strategy <1946 to September 18, 2020>.

## Data Availability

The datasets used and/or analyzed will be available from the corresponding author on reasonable request.

## Abbreviations

ADEs: Adverse drug events
ADRs: Adverse drug reactions
BMI: Body mass index
CIHI: Canadian Institute for Health Information
CKD: Chronic kidney disease
HRQoL: Health-related quality of life
OTC: Over the counter
PIMs: Potentially inappropriate medications
PRISMA-P 2015: Preferred reporting items for systematic reviews and meta-analyses for protocols 2015
RIMs: Renally inappropriate medications use
WHO: World Health Organization

## References

[1] Donaldson LJ, Kelley ET, Dhingra-Kumar N, Kieny MP, Sheikh A (2017). Medication without harm: who's third global patient safety challenge. Lancet.

[2] Duerden M, Payne R, Avery T (2013). Polypharmacy and medicines optimisation. King's Fund Report, November 2013.

[3] Mair A, Wilson M, Dreischulte T (2020). Addressing the challenge of polypharmacy. Annu Rev Pharmacol Toxicol.

[4] Jokanovic N, Tan EC, Dooley MJ, Kirkpatrick CM, Bell JS (2015). Prevalence and factors associated with polypharmacy in long-term care facilities: a systematic review. J Am Med Dir Assoc.

[5] Kantor ED, Rehm CD, Haas JS, Chan AT, Giovannucci EL (2015). Trends in prescription drug use among adults in the United States from 1999-2012. JAMA.

[6] Sarkar S (2017). Geriatric poly-pharmacy: a growing epidemic. How to prevent it?. Br J Med Med Res.

[7] Masnoon N, Shakib S, Kalisch-Ellett L, Caughey GE (2017). What is polypharmacy? A systematic review of definitions. BMC geriatrics.

[8] Gnjidic D, Hilmer SN, Blyth FM, Naganathan V, Waite L, Seibel MJ, McLachlan AJ, Cumming RG, Handelsman DJ, Le Couteur DG (2012). Polypharmacy cutoff and outcomes: five or more medicines were used to identify community-dwelling older men at risk of different adverse outcomes. J Clin Epidemiol.

[9] Barnett K, Mercer SW, Norbury M, Watt G, Wyke S, Guthrie B (2012). Epidemiology of multimorbidity and implications for health care, research, and medical education: a cross-sectional study. Lancet.

[10] National Center for Health Statistics (US). Health, United States, 2016: With Chartbook on Long-term Trends in Health. Hyattsville: National Center for Health Statistics (US); 2017. Report No.: 2017-1232. 28910066

[11] Qato DM, Wilder J, Schumm LP, Gillet V, Alexander GC (2016). Changes in prescription and over-the-counter medication and dietary supplement use among older adults in the United States, 2005 vs 2011. JAMA Intern Med.

[12] Proulx J, Hunt J (2015). Drug use among seniors on public drug programs in Canada, 2012. Healthcare Quarterly (Toronto).

[13] Wastesson JW, Morin L, Tan ECK, Johnell K (2018). An update on the clinical consequences of polypharmacy in older adults: a narrative review. Expert Opin Drug Saf.

[14] Davies LE, Spiers G, Kingston A, Todd A, Adamson J, Hanratty B (2020). Adverse outcomes of polypharmacy in older people: systematic review of reviews. J Am Med Dir Assoc.

[15] Khezrian M, McNeil CJ, Murray AD, Myint PK (2020). An overview of prevalence, determinants and health outcomes of polypharmacy. Ther Adv Drug Saf.

[16] Maher RL, Hanlon J, Hajjar ER (2014). Clinical consequences of polypharmacy in elderly. Expert Opin Drug Saf.

[17] Boyd CM, Darer J, Boult C, Fried LP, Boult L, Wu AW (2005). Clinical practice guidelines and quality of care for older patients with multiple comorbid diseases: implications for pay for performance. JAMA.

[18] Mangin D, Bahat G, Golomb BA, Mallery LH, Moorhouse P, Onder G, Petrovic M, Garfinkel D (2018). International group for reducing inappropriate medication use & polypharmacy (IGRIMUP): position statement and 10 recommendations for action. Drugs Aging.

[19] Garber J, Brownlee S (2019). Medication overload: America's Other Drug Problem. In.

[20] Garfinkel D, Ilhan B, Bahat G (2015). Routine deprescribing of chronic medications to combat polypharmacy. Ther Adv Drug Saf.

[21] Moffat K, Mercer SW (2015). Challenges of managing people with multimorbidity in today’s healthcare systems. BMC Fam Pract.

[22] Ahn R, Woodbridge A, Abraham A, Saba S, Korenstein D, Madden E, Boscardin WJ, Keyhani S. Financial ties of principal investigators and randomized controlled trial outcomes: cross sectional study. BMJ. 2017;356:i6770. 10.1136/bmj.i6770. 10.1136/bmj.i6770 PMC5241252 28096109

[23] Norris SL, Holmer HK, Ogden LA, Burda BU (2011). Conflict of interest in clinical practice guideline development: a systematic review. Plos One.

[24] Phillips KA, Veenstra D, Van Bebber S, Sakowski J (2003). An introduction to cost-effectiveness and cost-benefit analysis of pharmacogenomics. Pharmacogenomics.

[25] SA HLS: Under-reporting of adverse drug reactions: a systematic review. Drug Saf 2006, 29(5):385-396, doi: 10.2165/00002018-200629050-00003. 10.2165/00002018-200629050-00003 16689555

[26] Bourgeois FT, Shannon MW, Valim C, Mandl KD (2010). Adverse drug events in the outpatient setting: an 11-year national analysis. Pharmacoepidemiol Drug Saf.

[27] Leelakanok N, Holcombe AL, Lund BC, Gu X, Schweizer ML (2017). Association between polypharmacy and death: A systematic review and meta-analysis. J Am Pharm Assoc (2003).

[28] Tonelli M, Riella M (2014). Chronic kidney disease and the aging population. Brazilian J Nephrol.

[29] Nitta K, Okada K, Yanai M, Takahashi S (2013). Aging and chronic kidney disease. Kidney Blood Pressure Res.

[30] Titze S, Schmid M, Köttgen A, Busch M, Floege J, Wanner C, Kronenberg F, Eckardt K-U, Investigators GS, Eckardt K-U (2015). Disease burden and risk profile in referred patients with moderate chronic kidney disease: composition of the German Chronic Kidney Disease (GCKD) cohort. Nephrol Dial Transplan.

[31] USRDS (2018). United States Renal Data System. 2018 USRDS annual data report: Epidemiology of kidney disease in the United States.

[32] Mason NA (2011). Polypharmacy and medication-related complications in the chronic kidney disease patient. Curr Opin Nephrol Hypertens.

[33] Lea-Henry TN, Carland JE, Stocker SL, Sevastos J, Roberts DM (2018). Clinical Pharmacokinetics in Kidney Disease: Fundamental Principles. Clin J Am Soc Nephrol.

[34] Verbeeck RK, Musuamba FT (2009). Pharmacokinetics and dosage adjustment in patients with renal dysfunction. Eur J Clin Pharmacol.

[35] Schmidt IM, Hübner S, Nadal J, Titze S, Schmid M, Bärthlein B, Schlieper G, Dienemann T, Schultheiss UT, Meiselbach H (2019). Patterns of medication use and the burden of polypharmacy in patients with chronic kidney disease: the German Chronic Kidney Disease study. Clin Kidney J.

[36] Secora A, Alexander GC, Ballew SH, Coresh J, Grams ME (2018). Kidney function, polypharmacy, and potentially inappropriate medication use in a community-based cohort of older adults. Drugs Aging.

[37] Morin L, Laroche M-L, Texier G, Johnell K (2016). Prevalence of Potentially Inappropriate Medication Use in Older Adults Living in Nursing Homes: A Systematic Review. J Am Med Direct Assoc.

[38] Papaioannou A, Clarke JA, Campbell G, Bédard M (2000). Assessment of adherence to renal dosing guidelines in long-term care facilities. J Am Geriatr Soc.

[39] Chang F, O'Hare AM, Miao Y, Steinman MA (2015). Use of Renally Inappropriate Medications in Older Veterans: A National Study. J Am Geriatr Soc.

[40] Blix HS, Viktil KK, Moger TA, Reikvam A (2006). Use of renal risk drugs in hospitalized patients with impaired renal function--an underestimated problem?. Nephrol Dial Transplant.

[41] Laville SM, Metzger M, Stengel B, Jacquelinet C, Combe C, Fouque D, Laville M, Frimat L, Ayav C, Speyer E, Robinson BM, Massy ZA, Liabeuf S, on behalf of the Chronic Kidney Disease-Renal Epidemiology and Information Network (CKD-REIN) Study Collaborators (2018). Evaluation of the adequacy of drug prescriptions in patients with chronic kidney disease: results from the CKD-REIN cohort. Br J Clin Pharmacol.

[42] United Nations. World Population Prospects (The 2008 Revision).https://www.un.org/development/desa/pd/sites/www.un.org.development.desa.pd/files/files/documents/2020/Jan/un_2008_world_population_prospects-2008_revision_volume-ii.pdf. Accessed 2 July 2021.

[43] Moher D, Shamseer L, Clarke M, Ghersi D, Liberati A, Petticrew M, Shekelle P, Stewart LA, Group P-P (2015). Preferred reporting items for systematic review and meta-analysis protocols (PRISMA-P) 2015 statement. Syst Rev.

[44] Page MJ, McKenzie J, Bossuyt P, Boutron I, Hoffmann T, Mulrow C, Shamseer L, Tetzlaff J, Moher D (2020). Updating guidance for reporting systematic reviews: development of the PRISMA 2020 statement.

[45] Stroup DF, Berlin JA, Morton SC, Olkin I, Williamson GD, Rennie D, Moher D, Becker BJ, Sipe TA, Thacker SB (2000). Meta-analysis of observational studies in epidemiology: a proposal for reporting. Meta-analysis Of Observational Studies in Epidemiology (MOOSE) group. JAMA.

[46] Hoy D, Brooks P, Woolf A, Blyth F, March L, Bain C, Baker P, Smith E, Buchbinder R (2012). Assessing risk of bias in prevalence studies: modification of an existing tool and evidence of interrater agreement. J Clin Epidemiol.

[47] Campbell M, McKenzie JE, Sowden A, Katikireddi SV, Brennan SE, Ellis S, Hartmann-Boyce J, Ryan R, Shepperd S, Thomas J (2020). Synthesis without meta-analysis (SWiM) in systematic reviews: reporting guideline. BMJ.

[48] Higgins JP, Thompson SG, Deeks JJ, Altman DG (2003). Measuring inconsistency in meta-analyses. BMJ.

[49] Thompson SG, Higgins JP (2002). How should meta-regression analyses be undertaken and interpreted?. Stat Med.

[50] Higgins JP, Thompson SG (2002). Quantifying heterogeneity in a meta-analysis. Stat Med.

[51] Egger M, Davey Smith G, Schneider M, Minder C (1997). Bias in meta-analysis detected by a simple, graphical test. BMJ.

[52] Guyatt GH, Oxman AD, Vist GE, Kunz R, Falck-Ytter Y, Alonso-Coello P, Schünemann HJ (2008). GRADE: an emerging consensus on rating quality of evidence and strength of recommendations. BMJ.

