# Supplementary material for: Prevalence of polypharmacy and associated adverse health outcomes in adult patients with chronic kidney disease: protocol for a systematic review and meta-analysis
Source: Syst Rev. 2021 Jul 4;10:198. doi: 10.1186/s13643-021-01752-z (PMC8256607; doi:10.1186/s13643-021-01752-z)
Supplement: Supplementary file 2 — Additional file 2:. Ovid MEDLINE search terms and strategy <1946 to September 18, 2020>. [file 13643_2021_1752_MOESM2_ESM.docx]

**Additional File 2:** Ovid MEDLINE search terms and strategy <1946 to September 18, 2020>

| **#** | **Search term (number of hits)** |
| --- | --- |
| 1 | polypharmacy/ (5037) |
| 2 | Polypharmacy.mp. (10179) |
| 3 | polymedication*.mp. (237) |
| 4 | polymedicine*.mp. (12) |
| 5 | polydrug*.mp. (1573) |
| 6 | multiple prescription*.mp. (142) |
| 7 | Multiple prescrib*.mp. (129) |
| 8 | multiple medication*.mp. (1717) |
| 9 | multiple medicine.mp. (10) |
| 10 | multiple medicines.mp. (80) |
| 11 | multiple drug*.mp. (8207) |
| 12 | many medication*.mp. (673) |
| 13 | many medicine*.mp. (122) |
| 14 | many drug*.mp. (5242) |
| 15 | 1 or 2 or 3 or 4 or 5 or 6 or 7 or 8 or 9 or 10 or 11 or 12 or 13 or 14 (27231) |
| 16 | exp Renal Insufficiency, Chronic/ (115748) |
| 17 | Chronic Kidney disease*.mp. (54684) |
| 18 | chronic kidney insufficienc*.mp. (224) |
| 19 | chronic renal disease*.mp. (3892) |
| 20 | chronic renal insufficienc*.mp. (5079) |
| 21 | CKD.mp. (30427) |
| 22 | Renal fail*.mp. (90215) |
| 23 | Kidney fail*.mp. (100127) |
| 24 | or/16-23 (215749) |
| 25 | 15 and 24 (543) |
